# Global, regional, and national burden and trends of migraine among women of childbearing age from 1990 to 2021: insights from the Global Burden of Disease Study 2021

**DOI:** 10.1186/s10194-024-01798-z

**Published:** 2024-06-07

**Authors:** Jing Cen, Qian Wang, Lin Cheng, Qian Gao, Hongping Wang, Fengjun Sun

**Affiliations:** Department of Pharmacy, The First Affiliated Hospital of Army Medical University (Third Military Medical University), Chongqing, China

**Keywords:** Migraine, WCBA, Disease burden, DALYs, GBD 2021

## Abstract

**Background:**

Migraine, a neurological disorder with a significant female predilection, is the leading cause of disability-adjusted life years (DALYs) in women of childbearing age (WCBA). There is currently a lack of comprehensive literature analysis on the overall global burden and changing trends of migraines in WCBA.

**Methods:**

This study extracted three main indicators, including prevalence, incidence, and DALYs, related to migraine in WCBA from the Global Burden of Disease(GBD) database from 1990 to 2021. Our study presented point estimates with 95% uncertainty intervals (UIs). It evaluated the changing trends in the burden of migraine in WCBA using the estimated annual percentage change (EAPC) and percentage change.

**Results:**

In 2021, the global prevalence, incidence, and DALYs cases of migraine among WCBA were 493.94 million, 33.33 million, and 18.25 million, respectively, with percentage changes of 48%, 43%, and 47% compared to 1990. Over the past 32 years, global prevalence rates and DALYs rates globally have increased, with an EAPC of 0.03 (95% UI: 0.02 to 0.05) and 0.04 (95% UI: 0.03 to 0.05), while incidence rates have decreased with an EAPC of -0.07 (95% UI: -0.08 to -0.05). Among the 5 Socio-Demographic Index (SDI) regions, in 2021, the middle SDI region recorded the highest cases of prevalence, incidence, and DALYs of migraine among WCBA, estimated at 157.1 million, 10.56 million, and 5.81 million, respectively, approximately one-third of the global total. In terms of age, in 2021, the global incidence cases for the age group 15–19 years were 5942.5 thousand, with an incidence rate per 100,000 population of 1957.02, the highest among all age groups. The total number of migraine cases and incidence rate among WCBA show an increasing trend with age, particularly in the 45–49 age group.

**Conclusions:**

Overall, the burden of migraine among WCBA has significantly increased globally over the past 32 years, particularly within the middle SDI and the 45–49 age group. Research findings emphasize the importance of customized interventions aimed at addressing the issue of migraines in WCBA, thus contributing to the attainment of Sustainable Development Goal 3 set by the World Health Organization.

**Supplementary Information:**

The online version contains supplementary material available at 10.1186/s10194-024-01798-z.

## Introduction

Migraine is a disabling neurovascular disorder characterized by recurrent episodes of throbbing headaches, typically unilateral and of moderate to severe intensity. It represents a major global public health concern [1, 2]. The Global Burden of Disease(GBD) 2019 study estimates the global number of prevalent cases of migraine reached 1.1 billion, the prevalence to be around 14% (95% UI: 12.9 to 15.2) [3], exposing a growing population to the direct threat of migraine and presenting a challenge for societies to bear the increasing burden associated with the disease and healthcare systems. Indeed, migraine ranks second in terms of years lived with disability (YLDs) among all human diseases [4]. Migraine is more prevalent among females, particularly among women of childbearing age (WCBA) (15–49 years old), with the highest incidence rates observed in this demographic. Statistics indicate that annually, 21% to 28% of WCBA will experience migraines [5]. Furthermore, among females aged 18–44, the prevalence of migraine was highest, with a three-month prevalence of migraine or severe headache reported at 23.5% [6], with the peak onset of migraine without aura occurring between the ages of 14 and 17 [7].  

Compared to males, clinical manifestations of migraine in females are more severe. Women with migraines experience longer headaches, migraine symptoms, migraine-related disability, a higher burden of complications, and worsening with age [8]. Indeed, in women, migraine is among the top five most common causes of disability [9], especially as it is the leading cause of disability-adjusted life years (DALYs) among WCBA [3]. Due to the critical life stages of completing education, forming families, childbearing and raising children, and establishing professional careers, WCBA is more susceptible to experiencing migraines compared to females over the age of 49 [4, 10]. Furthermore, menstrual migraines and chronic migraines may potentially impact their pregnancy planning [11]. Unfortunately, existing research indicates that migraines during pregnancy are associated with adverse pregnancy outcomes for both the mother and the fetus, including gestational diabetes mellitus (GDM), hypertensive disorders of pregnancy (HDP), preterm birth, and low birth weight [12–14]. Additionally, the use of hormonal contraception (HC) can influence the burden of migraine in WCBA [15]. Therefore, it is important to assess the burden caused by migraines among WCBA to initiate precision intervention campaigns globally.  

It is worth noting that currently, there is only one study on the incidence and trends of migraine among women of childbearing age in China [16], and a comprehensive assessment of the burden of migraine among WCBA is largely unknown or unreported. Furthermore, the GBD 2019 report emphasized that headache disorders, especially migraine, hold a prominent position in the DALY rankings for the 10–49 age group globally yet receive little attention in global health policy debates [17]. The ideal scenario involves adhering to internationally recognized recommendations and establishing a tiered structure for healthcare service provision. However, the current reality is that in the majority of countries worldwide, healthcare system resources are insufficient, leading to a lack of standardized care for headache sufferers, inadequate education among healthcare personnel, and limited access for patients to highly specialized centres. For instance, in low- and middle-income countries, inadequate resources represent a primary challenge faced by healthcare professionals and patients alike. A fundamental issue in high-income countries is the low prioritization of care for headache patients [18]. Moreover, The ongoing COVID-19 pandemic makes further efforts to exacerbate these inequities [9]. Therefore, it is necessary to provide a comprehensive description and analysis of the overall disease status and changing trends of migraine among WCBA. Based on the latest data from the GBD 2021 study, we conducted an analysis of migraine incidence, prevalence, and DALYs among WCBA at the global, regional, and national levels from 1990 to 2021, comparing the distribution and changes in migraine burden across different age groups.

## Methods

### Data source and disease definition

The migraine data of WCBA analyzed in this study are derived from the GBD 2021, which provides the latest estimates of epidemiological data on the burden of 371 diseases and injuries across 21 GBD regions and 204 countries and territories from 1990 to 2021. All this data is accessible for free access through the Global Health Data Exchange (https://ghdx.healthdata.org/gbd-2021/sources) [19], with detailed information on the data, methodologies, and statistical modelling available in previous reports [20]. GBD 2021 classifies causes into four levels, ranging from Level 1 communicable, maternal, neonatal, nutritional diseases, to Level 4 latent tuberculosis infection. Migraine is classified as a Level 4 cause in GBD 2021 [19]. According to GBD 2021, migraine is defined as a disabling primary headache disorder characterized by recurrent moderate to severe pulsating unilateral head pain. In GBD 2021, we do not distinguish between migraine with and without aura as most epidemiological studies report on overall migraine only. A migraine is diagnosed if a patient's symptoms meet all five major diagnostic criteria as proposed by the International Classification of Headache Disorders^3rd^ edition (ICHD-3). According to the International Classification of Diseases(ICD), 9th and 10th editions, migraine is represented by codes 346–346.93 and G43-G43.919, respectively [19].

### Socio-demographic index (SDI)

The Socio-demographic Index (SDI), a comprehensive indicator introduced by the Institute for Health Metrics and Evaluation (IHME) in 2015, is designed to assess the development level of countries or regions, emphasizing the interconnection between social development and population health outcomes. In short, it is the geometric mean of 0 to 1 index of total fertility rate for those younger than 25 years old, mean education for those 15 years old and older, and lag-distributed income per capita. For GBD 2021 after calculating SDI, values were multiplied by 100 for a scale of 0 to 100, where 0 represents the lowest income and years of schooling, and highest fertility, Conversely, 100 indicates the highest income and years of schooling and lowest fertility. The 204 countries and territories were classified into five SDI regions: low, low-middle, middle, high-middle, and high in the GBD 2021 [19].

### Disability-adjusted life years

DALYs, a standard metric for quantifying burden, represent the total years of healthy life lost from the onset of a disease to death, encompassing both years of life lost (YLLs) and years lived with disability (YLDs), as expressed by the formula:

1 $$DALYs\hspace{0.17em}=\hspace{0.17em}YLLs\hspace{0.17em}+\hspace{0.17em}YLDs$$

Due to the lack of direct attribution of migraine-related deaths in the GBD estimates, YLLs are set to 0 in this study, leading to DALYs being equivalent to YLDs [19]. All calculations were conducted 500 times to generate draw-level estimates. The number of computations per process was reduced from 1000, as in previous GBD iterations, to 500 for GBD 2021 because simulation testing revealed the final estimates and their uncertainty was not affected by this reduction. Final estimates represent the mean estimate across 500 draws, and 95% uncertainty intervals (UIs) are represented by the 2.5th and 97.5th percentile values across the draws. Uncertainty was propagated at each step in the estimation process [19].

### Estimated annual percentage change and percentage change

The estimated annual percentage change (EAPC) is an effective and widely used indicator that has been extensively employed in previous studies to track trends in indicators such as prevalence and incidence rates over specific time periods [21]. This study aims to estimate the dynamic trends in the rates of prevalence, incidence, and DALYs of migraine from 1990 to 2021. The calculation of EAPC is based on fitting the natural logarithm rate of the regression model, with time as a variable, fitting the natural logarithm of each observation into a straight line, and calculating based on the slope of this line [22].

2 $$y\hspace{0.17em}=\hspace{0.17em}\alpha\hspace{0.17em}+\hspace{0.17em}\beta x\hspace{0.17em}+\hspace{0.17em}\varepsilon$$

3 $$EAPC\hspace{0.17em}=\hspace{0.17em}100\hspace{0.17em}\times\hspace{0.17em}(exp(\beta)-1)$$

x- year, y- the natural logarithm of rates (such as prevalence and incidence rates), α- the intercept, β- the slope, ε- the random error. The 95% confidence intervals (CIs) of the EAPC are also derived from this fitted model. The interpretation of trend results is based on the 95% CIs; a lower limit of 95% CIs greater than 0 indicates an upward trend, while an upper limit of 95% CIs lower than 0 suggests a downward trend. If the 95% CIs include 0, it indicates that there is no statistically significant difference in trend changes [23]. Furthermore, this study utilizes percentage change to reflect the variations in the cases of prevalence, incidence, and DALYs in 2021 compared to 1990.

4 $$Percentage\, change = (2021 \,cases -1990 \,cases)/ 1990 \,cases$$

Data cleaning, computation, and graph plotting were conducted using R software (version 4.3.1) in this study. Visualizations were created using the ggplot2 package, and final editing was done using Adobe Illustrator software (version CS5).

## Results

### Global level

**Fig. 1:**
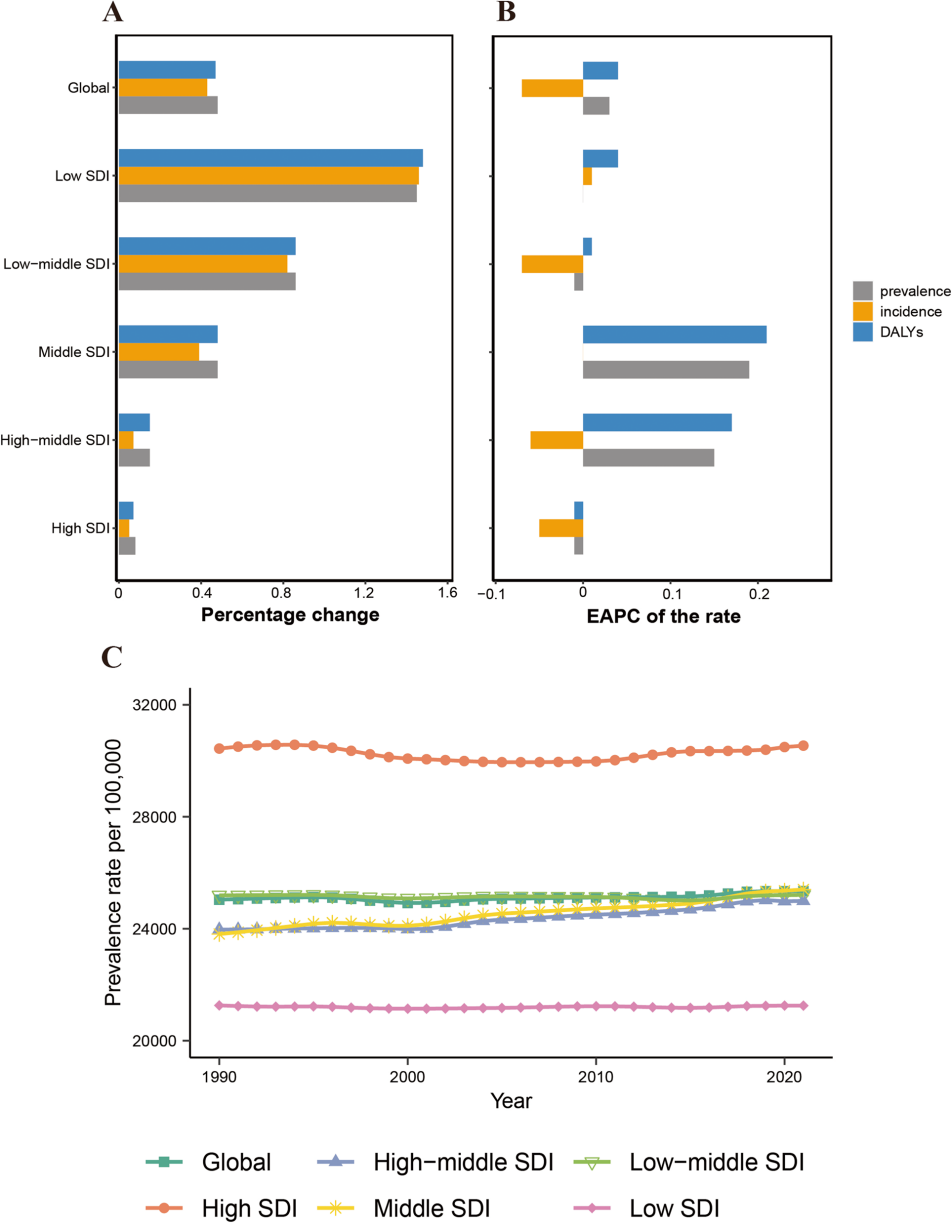
Temporal trend of migraine burden in WCBA in global and 5 territories. **A** Percentage change in cases of prevalent, incident, and DALYs in 1990 and 2021. **B** The EAPC of prevalence, incidence, and DALY rates from 1990 to 2021. **C** The rates of prevalence, incidence, and DALYs from 1990 to 2021. WCBA = Women of Childbearing Age, EAPC = Estimated Annual Percentage Change, DALYs = Disability-Adjusted Life Years

A significant increase in the cases of prevalent, incident, and DALYs of migraines related to WCBA was reported globally. For instance, the prevalent cases increased from 334.86 million in 1990 to 493.94 million in 2021, representing a percentage change of 48%, the incident cases increased from 23.36 million in 1990 to 33.33 million in 2021, with a percentage change of 43%, and the DALYs cases escalated from 12.38 million in 1990 to 18.25 million in 2021, showing a percentage change 47% (Table 1 and S1 and S2, Fig. 1A). However, the prevalence rates and DALY rates of migraines in WCBA globally increased from 1990 to 2021, with EAPC of 0.03 (95% CI: 0.02 to 0.05) and 0.04 (95% CI: 0.03 to 0.05), respectively. Conversely, the incidence rates exhibited a declining trend, with an EAPC of -0.07 (95% CI: -0.08 to -0.05). This indicates a continual escalation in the burden of migraines in WCBA worldwide (Tables 1 and S1 and S2, Fig. 1 and S1-S4).

**Table 1:** The prevalence of migraine cases and rates among WCBA in 1990 and 2021, and the trends from 1990 to 2021

Location	Prevalent cases			Prevalent rates			
	1990_millions(95% UI)	2021_millions(95% UI)	percentage change(100%)	1990_per 100 000(95% UI)	2021_per 100 000(95% UI)	EAPC(95% CI)	
Global	334.86 (283.29,391.36)	493.94 (420.68,577.87)	0.48	25,038.57 (21,183.1,29,263.57)	25,345.02 (21,586.18,29,652)	0.03 (0.02,0.05)	
Low SDI	23.75 (19.93,27.97)	58.3 (48.88,68.55)	1.45	21,260.92 (17,841.11,25,042.04)	21,252.06 (17,818.2,24,990.15)	0 (0,0.01)	
Low-middle SDI	68.75 (57.9,81.25)	127.63 (108.49,149.26)	0.86	25,192.45 (21,216.32,29,772)	25,209.45 (21,429.2,29,482.93)	-0.01 (-0.02,0)	
Middle SDI	106.45 (89.79,124.41)	157.1 (133.66,183.48)	0.48	23,810.87 (20,083.46,27,828.25)	25,400.85 (21,611.09,29,667.34)	0.19 (0.18,0.21)	
High-middle SDI	66.58 (56.71,77.94)	76.26 (64.92,89.36)	0.15	23,971.79 (20,417.21,28,059.28)	24,993.39 (21,275.12,29,284.74)	0.15 (0.14,0.17)	
High SDI	69 (58.96,80.7)	74.26 (63.88,86.75)	0.08	30,435.28 (26,008.41,35,595.94)	30,538.8 (26,269.54,35,676.44)	-0.01 (-0.04,0.02)	
High-income Asia Pacific	10.09 (8.58,11.83)	8.39 (7.12,9.91)	-0.17	22,066.42 (18,760.41,25,861.1)	22,060.64 (18,726.59,26,049.07)	-0.05 (-0.06,-0.03)	
High-income North America	26.12 (22.17,30.37)	28.88 (24.66,33.82)	0.11	35,119.92 (29,805.93,40,839.95)	34,371 (29,349.95,40,249.23)	-0.04 (-0.11,0.03)	
Western Europe	33.95 (28.91,40.12)	33.44 (28.52,39.35)	-0.02	35,534.54 (30,254.79,41,990.49)	35,891.2 (30,606.35,42,232.51)	0.03 (-0.01,0.07)	
Australasia	1.31 (1.09,1.55)	1.78 (1.47,2.1)	0.36	24,466.52 (20,216.73,28,857.37)	24,602.47 (20,395.35,29,050.75)	0.01 (0,0.01)	
Andean Latin America	1.71 (1.46,2.01)	3.4 (2.84,4.02)	0.99	18,063.62 (15,434.89,21,187.75)	19,480.42 (16,276.01,23,011.3)	0.27 (0.21,0.33)	
Tropical Latin America	12.87 (10.97,15.06)	19.41 (16.57,22.73)	0.51	32,252.62 (27,499.77,37,750.09)	32,026.13 (27,335.54,37,502.93)	-0.01 (-0.04,0.02)	
Central Latin America	10.88 (9.27,12.91)	17.98 (15.29,21.27)	0.65	25,967.99 (22,109.21,30,810.34)	26,366.88 (22,422.17,31,190.31)	0.05 (0.05,0.06)	
Southern Latin America	2.65 (2.23,3.18)	3.88 (3.25,4.66)	0.46	21,385.84 (18,003.07,25,670.32)	22,234.3 (18,653.23,26,727.43)	0.17 (0.15,0.19)	
Caribbean	2.39 (1.97,2.85)	3.08 (2.56,3.67)	0.29	25,638.57 (21,155.36,30,539.64)	25,633.49 (21,283.19,30,523)	0 (0,0)	
Central Europe	7.92 (6.67,9.32)	6.77 (5.74,8)	-0.15	25,775.43 (21,719.78,30,355.85)	26,302.22 (22,306.82,31,077.77)	0.07 (0.06,0.09)	
Eastern Europe	14.37 (12.39,16.77)	12.8 (10.99,14.95)	-0.11	25,996.49 (22,409.86,30,329.99)	26,538.2 (22,779.48,30,995.68)	0.08 (0.06,0.09)	
Central Asia	4.1 (3.37,4.92)	6.05 (4.99,7.27)	0.48	24,435.98 (20,115.2,29,327.04)	24,927.57 (20,552.19,29,962.95)	0.05 (0.04,0.06)	
North Africa and Middle East	21.24 (17.75,25.4)	44.71 (38.27,52.91)	1.10	27,184.48 (22,723.51,32,509.67)	28,057.22 (24,017.02,33,207.22)	0.13 (0.11,0.14)	
South Asia	63.61 (53.58,74.67)	123.59 (104.34,144.41)	0.94	24,956.9 (21,021.77,29,296.3)	25,011.41 (21,116.26,29,225.28)	-0.02 (-0.04,0)	
Southeast Asia	34.09 (28.74,40.52)	51.29 (43.39,60.87)	0.50	28,365.96 (23,910.45,33,714.38)	27,993.98 (23,686.49,33,223.87)	-0.05 (-0.07,-0.03)	
East Asia	64.7 (54.46,75.91)	70.8 (59.76,84.05)	0.09	19,408.31 (16,335.35,22,770.67)	21,395.48 (18,060.7,25,401.43)	0.32 (0.29,0.36)	
Oceania	0.37 (0.3,0.44)	0.83 (0.68,1)	1.24	23,603.1 (19,351.19,28,411.18)	23,825.63 (19,726.56,28,749.33)	0.03 (0.02,0.03)	
Western Sub-Saharan Africa	10.63 (8.83,12.71)	29.18 (24.33,34.74)	1.75	24,389.1 (20,254.41,29,156.85)	24,339.59 (20,297.31,28,976.86)	0 (-0.01,0)	
Eastern Sub-Saharan Africa	6.47 (5.38,7.66)	16.26 (13.49,19.15)	1.51	15,001.75 (12,463.82,17,760.82)	15,183.66 (12,592.46,17,884)	0.06 (0.05,0.07)	
Central Sub-Saharan Africa	2.54 (2.1,3.07)	6.73 (5.56,8.1)	1.65	20,575.87 (16,971.16,24,797.12)	20,609.53 (17,031.76,24,800.79)	0 (0,0.01)	
Southern Sub-Saharan Africa	2.82 (2.35,3.34)	4.69 (3.97,5.54)	0.66	21,211.65 (17,693.56,25,101.78)	21,597.45 (18,305.98,25,510.89)	0.06 (0.05,0.06)	

*Abbreviations:* *WCBA* Women of Childbearing Age, *EAPC* Estimated Annual Percentage Change, *CI* Confidence Intervals, *UI* Uncertainty Intervals, *SDI* Socio-Demographic Index

## SDI regional level 

**Fig. 2:**
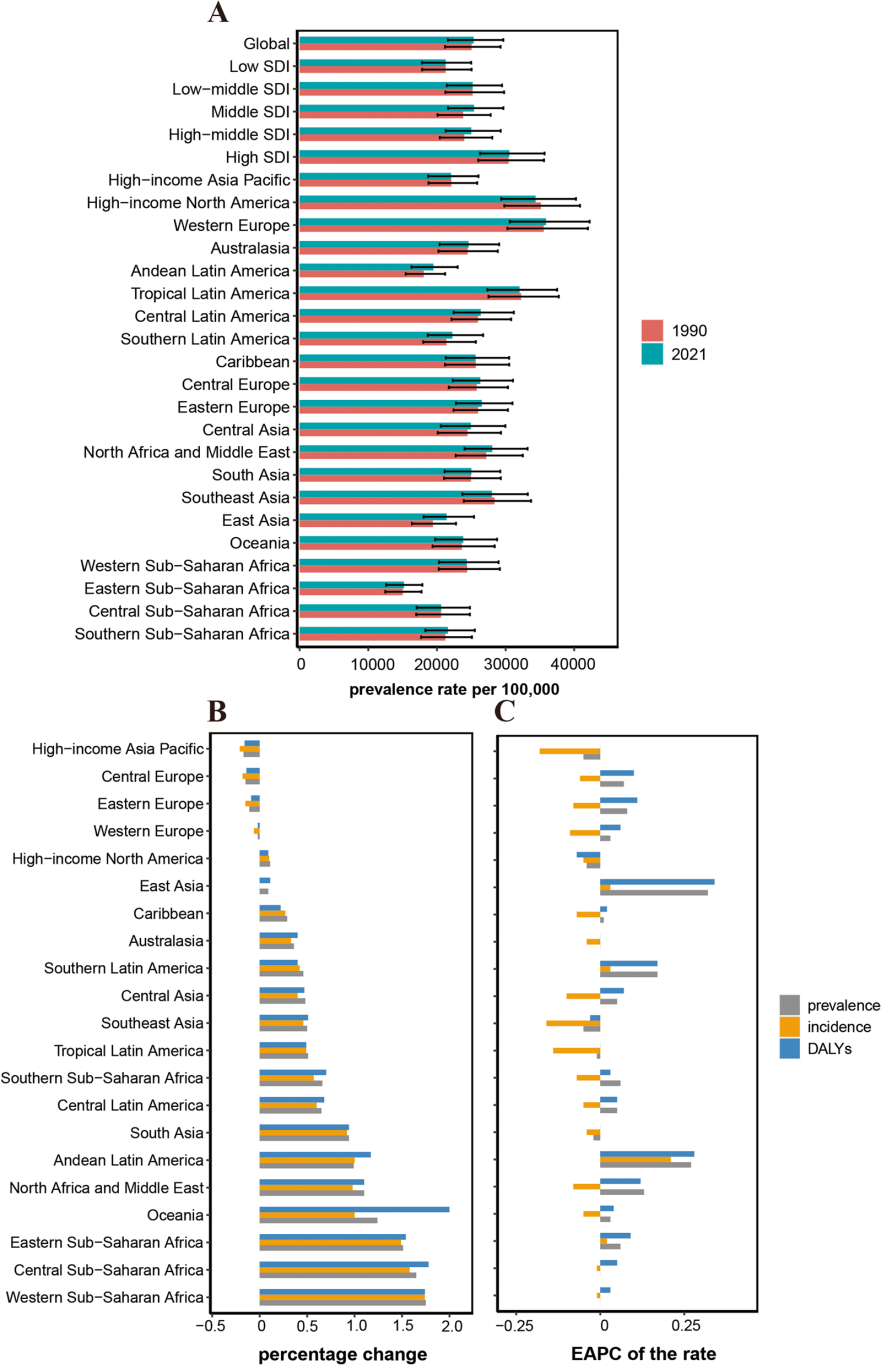
Temporal trend of migraine burden in WCBA in regions. **A** Prevalence rate per 100,000 population in 1990 and 2021. **B** Percentage change in cases of prevalent, incident, and DALYs in 1990 and 2021. **C** EAPC of rates of prevalent, incident, and DALYs from 1990 to 2021. WCBA = Women of Childbearing Age, EAPC = Estimated Annual Percentage Change, DALYs = Disability-Adjusted Life Years

In 2021, the highest absolute cases of prevalent, incident, and DALY of migraines in WCBA were observed in the middle SDI, with 157.1 million (95% UI: 133.66 to 183.48), 10.56 million (95% UI: 9.01 to 12.59), and 5.81 million (95% UI: 0.65 to 12.65), respectively. Furthermore, these cases accounted for approximately one-third of the global total. In 2021, the highest rates of prevalence, incidence, and DALY were shown in the high SDI regions (Tables 1 and S1 and S2, Fig. 1C and 2A and S1 − S4). The cases of prevalence, incidence, and DALYs increased gradually with decreasing SDI, with the low SDI region showing the largest percentage change (approximately 150%). In contrast, the high SDI region exhibited the smallest percentage change (approximately 5%-8%) (Fig. 1A). Notably, from 1990 to 2021, the middle SDI region demonstrated a rapid increase in both prevalence rates and DALY rates, with EAPC of 0.19 (95% CI: 0.18 to 0.21) and 0.21 (95% CI: 0.19 to 0.22), respectively (Tables 1 and S3 and S4, Fig. 1B). Furthermore, in 2021, the middle SDI region also exhibited relatively higher rates of prevalence, incidence, and DALYs. Therefore, the middle SDI region had the highest cases of prevalence, incidence, and DALYs of migraines in WCBA, with the most rapid increase in these rates.

### GBD regional level

The absolute numbers of prevalence, incidence, and DALYs of migraines in WCBA increased over time and were observed in the majority of regions. Only a few regions, specifically Eastern Europe, Central Europe, and the high-income Asia Pacific exhibited a decrease, all of which are from high SDI/high − middle SDI regions. Over the past 32 years, consistent increases in both prevalence rates and DALYs rates have been observed in the majority of regions, with the largest increases in East Asia and Andean Latin America; the EAPC for prevalence rates were 0.32 (95% CI: 0.29 to 0.36) and 0.27 (95% CI: 0.21 to 0.33) and for DALY rates of 0.34 (95% CI: 0.31 to 0.38) and 0.28 (95% CI: 0.22 to 0.34). In contrast, decreases in both prevalence rates and DALY rates were observed in high-income North America and Southeast Asia; the EAPC for prevalence rates were -0.04 (95% CI: -0.11 to 0.03) and -0.05 (95% CI: -0.07 to -0.03) and for DALY rates of -0.07 (95% CI: -0.13 to 0) and -0.03 (95% CI: -0.05 to -0.01). The incidence rates of migraine in WCBA showed an opposite trend to the prevalence rates, decreasing over time in 17 regions. In the high-income Asia Pacific region, a significant downward trend is observed in both numbers and rates of prevalence, incidence, and DALYs of migraine, indicating a decreasing burden of migraine within the region. This trend could be closely associated with improvements in healthcare and economic conditions (Tables 1 and S1 and S2, Fig. 2 and S3 and S4).

### Countries level

**Fig. 3:**
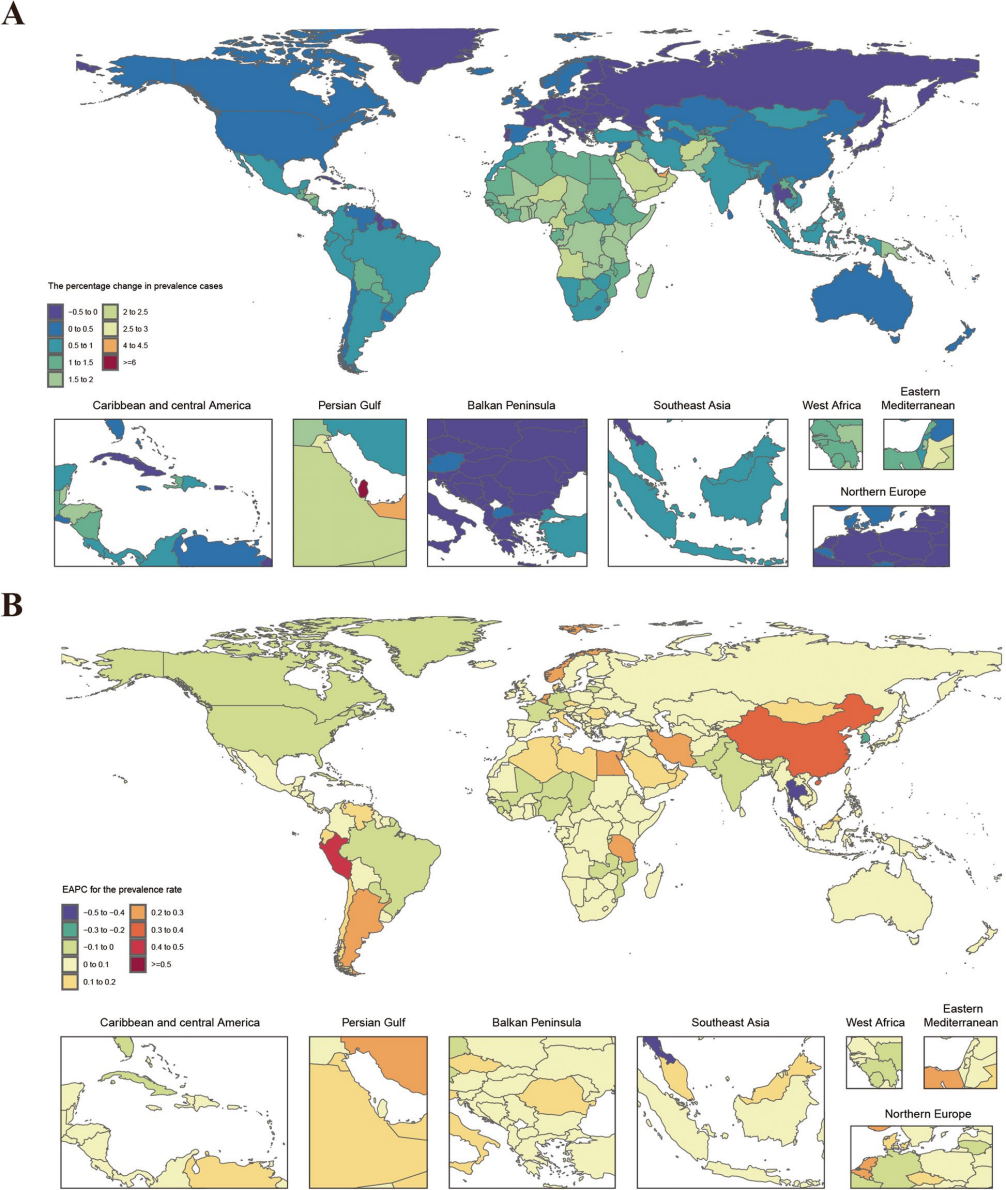
Temporal trend of migraine burden in WCBA globally. **A** Percentage change in prevalent cases across 204 countries in 1990 and 2021. **B** EAPC in prevalent rates across 204 countries from 1990 to 2021. WCBA = Women of Childbearing Age, EAPC = Estimated Annual Percentage Change

Approximately 76% of countries exhibited an increasing trend in the cases of prevalence, incidence, and DALYs of migraine among WCBA from 1990 to 2021. The highest percentage changes were observed in Qatar and the United Arab Emirates, both classified as high SDI regions, ranging from 350 to 620%. This contrasts with the notably lower percentage changes observed in other high SDI regions. Additionally, only 24% of countries exhibited a decrease in the cases of prevalence, incidence, and DALYs, such as Georgia (middle SDI), Virgin Islands and Latvia (high SDI), with percentage changes ranging from 39 to 44%. This demonstrates a polarizing trend in the increase of migraine cases among WCBA in countries within the high SDI regions. Over the past 32 years, the majority of countries showed upward trends in prevalence rates and DALY rates; the highest increase was observed in Singapore from the high SDI region, with an EAPC of 0.53 (95% CI: 0.4 to 0.67) and 0.5 (95% CI: 0.38 to 0.62), contrary to the global trend in high SDI regions. Only a few countries showed an upward trend in incidence rates; the most significant increases in incidence rates were observed in Peru and Singapore, with EAPC 0.35 (95% CI: 0.26 to 0.45 and 0.2 (95% CI: 0.11 to 0.29). The majority of countries showed a decreasing trend in incidence rates, with South Korea and Thailand exhibited the most significant declines, with EAPC -0.39 (95% CI: -0.44 to -0.34) and -0.37 (95% CI: -0.42 to -0.33), respectively(Tables S3-S5, Fig. 3 and S5-S8).

### Age patterns

**Fig. 4:**
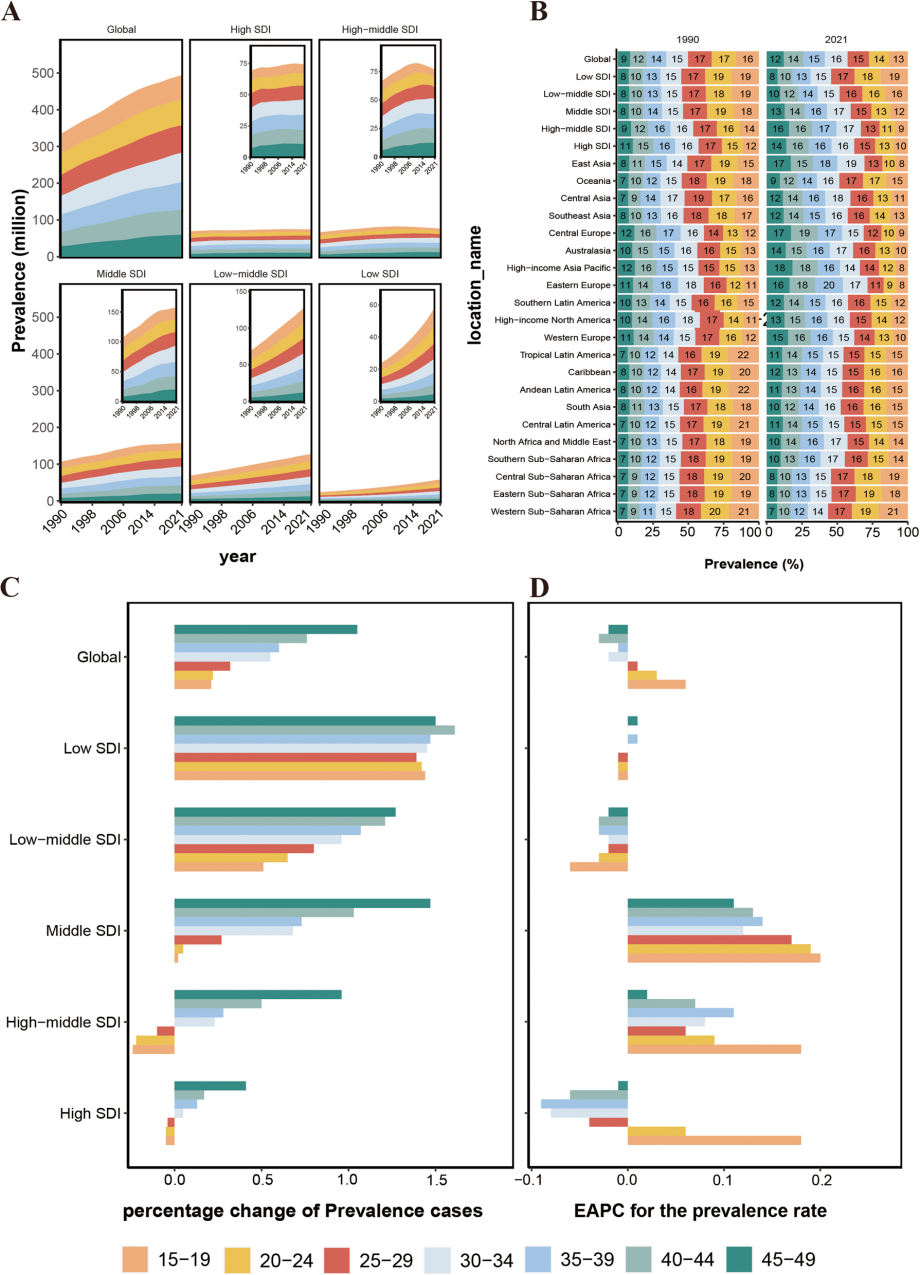
Temporal trend of migraine burden in WCBA by age pattern in different regions. **A** Prevalent cases of 7 age groups (15–49 years, 5-year intervals) from 1990 to 2021 globally and in 5 territories (low to high SDI). **B** The distribution of prevalent cases across 7 age groups as percentages globally, in 5 territories, and 21 GBD regions in 1990 and 2021. **C** Percentage change in prevalent cases of 7 age groups globally and in 5 territories in 1990 and 2021. **D** EAPC of prevalent rates of 7 age groups globally and in 5 territories from 1990 to 2021. WCBA = Women of Childbearing Age, EAPC = Estimated Annual Percentage Change, SDI = Socio-Demographic Index, GBD = Global Burden of Disease

The detailed trends of migraine cases over the past 32 years in both the global and the five SDI regions are illustrated in Fig. 4A S9 and S10. The percentage change in the number of prevalence, incidence, and DALYs of migraine among WCBA global increases with age, with the lowest percentage change being exhibited by the 15–19 age group at 21%, 18%, and 21%. Meanwhile, the 45–49 age group exhibited the highest percentage change, with 105%, 111%, and 104%, respectively, approximately 5–6 times higher than the increments in the 15–19 age group. Across the five SDI regions, the percentage change in migraine cases among WCBA shows diverse trends with age. Consistently, the highest percentage changes are observed in low SDI regions across all age groups, remaining relatively stable at around 150%. Conversely, in low-middle SDI to high SDI regions, the percentage change of migraine cases gradually increased with increasing age. For instance, the percentage change of prevalent cases in the middle SDI region rises from 0.02 in the 15–19 age group to 1.47 in the 45–49 age group. However, with improved economic conditions, the percentage change in each age group gradually decreases. For example, the percentage change of prevalent cases in the 45–49 age group declines from 1.27 in low-middle SDI to 0.41 in high SDI regions. Remarkably, in middle SDI regions, the surge in migraine cases in the 45–49 age group reaches a peak of 147%, surpassing all other age groups and SDI regions in terms of percentage change( Tables 2 and S8 and S9, Fig. 4C and S11 and S12). The trend analysis of global incidence rates among WCBA from 1990 to 2021 reveals a decreasing trend in the 15–19 and 20–24 age groups, with EAPC being -0.04 (95% CI: -0.08 to -0.01) and -0.04 (95% CI: -0.05 to -0.02), respectively. In contrast, the incidence rates in the 25–49 age group demonstrate an increasing trend. This indicates a rising trend in the cases of prevalence, incidence, DALYs, and incidence rates of migraine among WCBA over the past 32 years globally with age, with the highest increase observed in the 45–49 age group(Table S6, Fig. 4C and S13).

**Table 2:** The prevalence of migraine cases and rates among WCBA in 1990 and 2021, and the trends in age patterns from 1990 to 2021

Location	Age(years)	Prevalent cases			Prevalent rates		
		1990_thousands(95% UI)	2021_thousands(95% UI)	percentage change(100%)	1990_per 100 000(95% UI)	2021_per 100 000(95% UI)	EAPC(95% CI)
Global	15–19	54,179.88 (40,823.53,68,589.92)	65,470.85 (49,518.82,83,378.72)	0.21	21,202.21 (15,975.47,26,841.29)	21,561.29 (16,307.86,27,458.83)	0.06 (0.04,0.08)
	20–24	58,004.07 (44,902.44,72,364.3)	70,740.59 (54,626.52,88,391.02)	0.22	23,759.02 (18,392.47,29,641.1)	24,081.74 (18,596.13,30,090.35)	0.03 (0.01,0.05)
	25–29	56,480.98 (45,180.94,69,813.59)	74,634.15 (59,246.07,92,560.68)	0.32	25,661.56 (20,527.5,31,719.09)	25,648.6 (20,360.36,31,809.19)	0.01 (-0.01,0.03)
	30–34	51,318.79 (40,917.96,64,207.01)	79,725.64 (63,426.23,100,522.63)	0.55	26,994.4 (21,523.42,33,773.78)	26,670.26 (21,217.69,33,627.38)	-0.02 (-0.04,0.01)
	35–39	47,237.32 (38,499.45,58,293.84)	75,735.39 (61,217.26,93,691.79)	0.60	27,233.45 (22,195.86,33,607.8)	27,262.29 (22,036.23,33,726.02)	-0.01 (-0.04,0.01)
	40–44	38,673.19 (31,333.83,48,221.21)	68,121.81 (55,223.57,85,072.98)	0.76	27,579.29 (22,345.32,34,388.34)	27,458.52 (22,259.5,34,291.2)	-0.03 (-0.04,-0.01)
	45–49	28,962.09 (23,360.7,36,006.35)	59,509.42 (47,451.46,74,183.85)	1.05	25,449.84 (20,527.73,31,639.84)	25,254.1 (20,137.05,31,481.51)	-0.02 (-0.05,0)
	15–49	334,856.32 (283,294.75,391,359.76)	493,937.86 (420,683.36,577,874.6)	0.48	25,038.57 (21,183.1,29,263.57)	25,345.02 (21,586.18,29,652)	0.03 (0.02,0.05)
Low SDI	15–19	4625.24 (3427.51,5954.72)	11,302.85 (8341.55,14,589.78)	1.44	18,406.13 (13,639.76,23,696.76)	18,331.47 (13,528.71,23,662.36)	-0.01 (-0.01,0)
	20–24	4430.82 (3379.02,5610.63)	10,739.85 (8201.8,13,515)	1.42	20,398.99 (15,556.62,25,830.72)	20,366.75 (15,553.66,25,629.45)	-0.01 (-0.02,0)
	25–29	4038.41 (3161.84,5063.96)	9636.02 (7528.74,12,061.81)	1.39	21,801.69 (17,069.46,27,338.21)	21,875.66 (17,091.73,27,382.68)	-0.01 (-0.02,0)
	30–34	3490.25 (2751.43,4476.53)	8537.51 (6754.26,11,007.94)	1.45	22,945.67 (18,088.48,29,429.71)	23,000.75 (18,196.52,29,656.31)	0 (-0.01,0.01)
	35–39	3007.69 (2422,3744.69)	7430.57 (5932.5,9209.36)	1.47	23,343.24 (18,797.63,29,063.26)	23,318.34 (18,617.17,28,900.48)	0.01 (0,0.02)
	40–44	2333.74 (1889.68,2934.4)	6100.28 (4937.16,7697.64)	1.61	23,537.08 (19,058.44,29,595.02)	23,354.87 (18,901.89,29,470.36)	0 (-0.02,0.01)
	45–49	1819.18 (1449.89,2306.3)	4549.66 (3604.2,5772.17)	1.50	21,913.44 (17,465.09,27,781.21)	21,907.66 (17,355.05,27,794.31)	0.01 (-0.01,0.02)
	15–49	23,745.33 (19,925.91,27,968.29)	58,296.74 (48,877.29,68,550.75)	1.46	21,260.92 (17,841.11,25,042.04)	21,252.06 (17,818.2,24,990.15)	0 (0,0.01)
Low-middle SDI	15–19	13,289.53 (9966.98,16,949.98)	20,122.2 (15,174.65,25,634.72)	0.51	22,619.77 (16,964.54,28,850.12)	22,258.79 (16,785.9,28,356.62)	-0.06 (-0.07,-0.04)
	20–24	12,652.54 (9738.92,15,881.49)	20,935.5 (16,263.38,26,144.19)	0.65	24,259.76 (18,673.23,30,450.89)	24,061.29 (18,691.6,30,047.68)	-0.03 (-0.04,-0.02)
	25–29	11,492.82 (9125.25,14,386.59)	20,704.26 (16,311.82,25,736.56)	0.80	25,606.3 (20,331.3,32,053.7)	25,483.13 (20,076.84,31,676.96)	-0.02 (-0.03,-0.01)
	30–34	10,063.7 (7990.71,12,646.89)	19,765.71 (15,827.42,24,910.39)	0.96	26,876.87 (21,340.59,33,775.73)	26,743.67 (21,415.03,33,704.6)	-0.02 (-0.03,-0.01)
	35–39	8729.83 (7059.46,10,916.82)	18,043.32 (14,538.09,22,508.79)	1.07	27,251.71 (22,037.34,34,078.77)	27,097.42 (21,833.28,33,803.65)	-0.03 (-0.05,-0.02)
	40–44	7063 (5688.81,8892.97)	15,639.26 (12,669.45,19,652.95)	1.21	27,223.5 (21,926.83,34,276.9)	27,113 (21,964.39,34,071.34)	-0.03 (-0.04,-0.02)
	45–49	5462.92 (4337.5,6864.76)	12,417.83 (9861.78,15,601.82)	1.27	25,168.8 (19,983.73,31,627.34)	25,118.46 (19,948.15,31,558.96)	-0.02 (-0.03,-0.01)
	15–49	68,754.35 (57,902.85,81,252.7)	127,628.08 (108,489.8,149,263.45)	0.86	25,192.45 (21,216.32,29,772)	25,209.45 (21,429.2,29,482.93)	-0.01 (-0.02,0)
Middle SDI	15–19	19,077.26 (14,448.78,24,299.24)	19,431.99 (14,654.27,24,775.48)	0.02	20,690.22 (15,670.41,26,353.72)	22,122.52 (16,683.28,28,205.86)	0.2 (0.16,0.24)
	20–24	19,978.29 (15,561.14,24,945.82)	21,061.11 (16,329.36,26,292.4)	0.05	22,617.25 (17,616.64,28,240.95)	24,301.04 (18,841.39,30,337.08)	0.19 (0.16,0.22)
	25–29	18,319.05 (14,619.52,22,595.75)	23,195.63 (18,394.12,28,784.42)	0.27	24,455.44 (19,516.66,30,164.73)	25,602.64 (20,302.88,31,771.38)	0.17 (0.13,0.2)
	30–34	15,624.43 (12,393.19,19,548.83)	26,193.15 (20,903.19,32,905.08)	0.68	26,023.84 (20,641.93,32,560.26)	26,523.85 (21,167.1,33,320.52)	0.12 (0.08,0.17)
	35–39	14,372.92 (11,684.64,17,839.12)	24,897.56 (20,085.86,30,838.91)	0.73	25,929.14 (21,079.41,32,182.28)	27,166.61 (21,916.4,33,649.43)	0.14 (0.09,0.18)
	40–44	10,947.53 (8844.87,13,699.61)	22,236.24 (17,919.73,27,841.53)	1.03	25,907.56 (20,931.57,32,420.41)	27,166.79 (21,893.17,34,014.98)	0.13 (0.1,0.15)
	45–49	8130.3 (6562.23,10,109.66)	20,080.18 (15,887.97,25,168.05)	1.47	23,988.11 (19,361.6,29,828.13)	24,756.39 (19,587.92,31,029.11)	0.11 (0.07,0.15)
	15–49	106,449.77 (89,785.87,124,410.03)	157,095.85 (133,657.46,183,482.69)	0.48	23,810.87 (20,083.46,27,828.25)	25,400.85 (21,611.09,29,667.34)	0.19 (0.18,0.21)
High-middle SDI	15–19	9173.76 (6819.44,11,639.56)	7011.27 (5206.76,8917.1)	-0.24	19,370.16 (14,399.08,24,576.62)	20,361.41 (15,120.94,25,896.14)	0.18 (0.12,0.24)
	20–24	10,724.67 (8316.91,13,473.78)	8312.26 (6345.74,10,485.96)	-0.22	22,277.65 (17,276.16,27,988.18)	23,351.84 (17,827.22,29,458.45)	0.09 (0.04,0.13)
	25–29	11,136.84 (8855.95,13,907.18)	10,030 (7919.11,12,510.87)	-0.10	24,345.08 (19,359.07,30,401.04)	24,882.74 (19,645.98,31,037.36)	0.06 (0.02,0.11)
	30–34	10,735.25 (8562.63,13,477.84)	13,227.68 (10,522.68,16,684.28)	0.23	25,714.17 (20,510.07,32,283.5)	25,689.42 (20,436.06,32,402.49)	0.08 (0.03,0.12)
	35–39	10,357.72 (8442.77,12,798.6)	13,213.01 (10,676.27,16,269.25)	0.28	26,224.15 (21,375.8,32,404.08)	26,665.52 (21,546.06,32,833.39)	0.11 (0.09,0.14)
	40–44	8281.67 (6703.15,10,362)	12,398.57 (9995.2,15,437.08)	0.50	26,937.31 (21,802.92,33,703.85)	27,238.05 (21,958.15,33,913.25)	0.07 (0.05,0.1)
	45–49	6172.67 (4995.53,7667.85)	12,069.03 (9521.18,15,114.42)	0.96	25,176.68 (20,375.43,31,275.15)	25,024.94 (19,742.01,31,339.51)	0.02 (-0.02,0.05)
	15–49	66,582.59 (56,709.6,77,935.75)	76,261.83 (64,916.33,89,355.91)	0.15	23,971.79 (20,417.21,28,059.28)	24,993.39 (21,275.12,29,284.74)	0.15 (0.14,0.17)
High SDI	15–19	7962.75 (5873.25,10,003.33)	7551.7 (5622.76,9660.16)	-0.05	24,985.88 (18,429.37,31,388.92)	25,958.14 (19,327.6,33,205.72)	0.18 (0.11,0.24)
	20–24	10,165.13 (7884.98,12,814.59)	9636.71 (7486.93,12,105.73)	-0.05	30,273.02 (23,482.43,38,163.42)	30,569.78 (23,750.22,38,402.06)	0.06 (0.01,0.11)
	25–29	11,442.31 (9224.22,14,042.27)	11,009.58 (8853.94,13,558.37)	-0.04	31,920.68 (25,732.86,39,173.79)	31,857.2 (25,619.67,39,232.34)	-0.04 (-0.08,0.01)
	30–34	11,354.76 (9118.6,14,217.62)	11,939.37 (9557.71,14,946.21)	0.05	31,999.12 (25,697.34,40,067.04)	31,895.14 (25,532.72,39,927.68)	-0.08 (-0.12,-0.04)
	35–39	10,721.89 (8742.78,13,156.99)	12,089.45 (9836.91,14,838.23)	0.13	32,063.85 (26,145.32,39,346.03)	31,871.13 (25,932.82,39,117.66)	-0.09 (-0.11,-0.06)
	40–44	10,006.15 (8058.42,12,379.28)	11,688.76 (9516,14,458.99)	0.17	32,047.32 (25,809.18,39,647.89)	31,839.47 (25,920.99,39,385.38)	-0.06 (-0.08,-0.05)
	45–49	7345.83 (5888.22,9090.04)	10,341.87 (8253.47,12,943.78)	0.41	29,073.24 (23,304.31,35,976.45)	28,802.58 (22,986.29,36,049.02)	-0.01 (-0.03,0.01)
	15–49	68,998.81 (58,962.81,80,698.39)	74,257.45 (63,876.43,86,750.03)	0.08	30,435.28 (26,008.41,35,595.94)	30,538.8 (26,269.54,35,676.44)	-0.01 (-0.04,0.02)

*Abbreviations:* *WCBA* Women of Childbearing Age, *EAPC* Estimated Annual Percentage Change, *CI* Confidence Intervals, *UI* Uncertainty Intervals, *SDI* Socio-Demographic Index

In 2021, 5942.5 thousand (95% UI: 3496.17 to 8946.56) incidence cases of migraine among WCBA in the aged 15–19 were reported globally, with incidence rates per 100,000 of 1957.02 (95% UI: 1151.38 to 2946.34), which were the highest among all age groups (Table S6). Additionally, the fastest increases in the prevalence rates and DALY rates of migraine among WCBA globally were observed in the 15–19 age group from 1990 to 2021, with an EAPC of 0.06 (95% CI:0.04 to 0.08) and 0.07 (95% CI:0.05 to 0.09). This indicates that the new burden of migraine among WCBA was most severe in the 15–19 age group globally in 2021 (Tables 2 and S9, Fig. 4D and S14).

From 1990 to 2021, the proportion of migraine cases among WCBA globally, in terms of prevalence, incidence, and DALYs, decreased across the age groups of 15–19, 20–24, and 25–29 years, while increasing in the age groups of 30–34, 35–39, 40–44, and 45–49 years. In the five SDI regions, the trends of the proportion from low-middle SDI to high SDI regions across age groups mirror the global trend, with no change observed in the low SDI region. Among the 21 regions, East Asia showed the most significant increase in the proportion of migraine cases among WCBA in the 45–49 age group. The proportion of prevalence cases increased from 8% in 1990 to 17% in 2021, the proportion of incidence cases increased from 5 to 12%, and the proportion of DALY cases increased from 8 to 17%. Moreover, this region also experienced the most significant decrease in the proportion of cases in the 15–19 age group. Therefore, there is a trend of transition in the proportion of migraine numbers among WCBA from younger age groups to older age groups (Fig. 4B and S15 and S16).

### The association between migraine burden and SDI

**Fig. 5:**
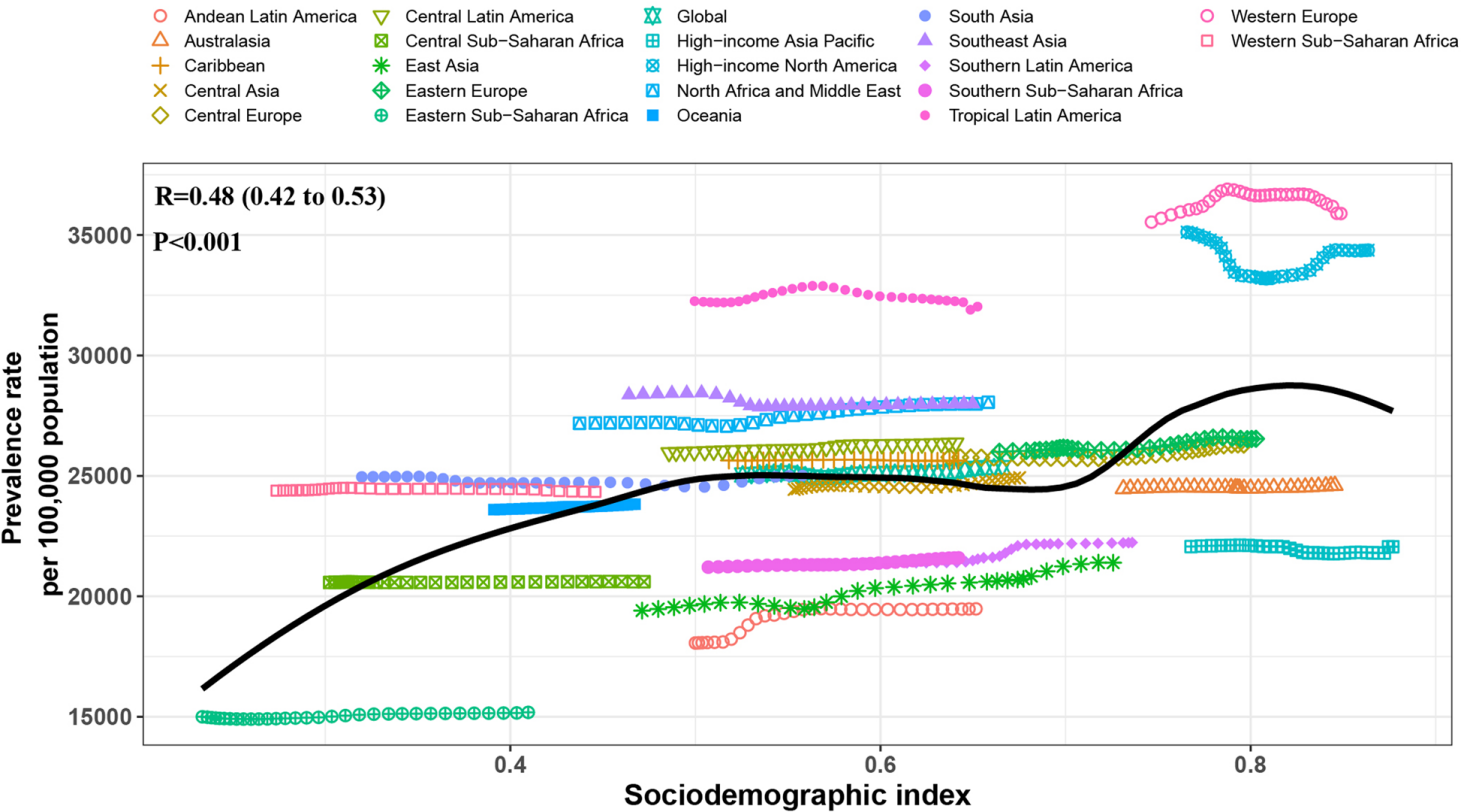
The associations between the SDI and prevalent rates per 100,000 population of migraine in WCBA across 21 GBD regions. WCBA = Women of Childbearing Age, SDI = Socio-Demographic Index, GBD = Global Burden of Disease

In 2021, there was a positive correlation between prevalence rates, incidence rates, and DALYs rates of migraine in WCBA and the SDI. With the improvement of the economy, the overall disease burden is on the rise. Overall, the global burden of migraine is slightly higher than expected. Across 21 regions, the burden of migraine in WCBA remains relatively stable when the SDI value falls between 0.5 and 0.7. The burden of migraine reaches its peak when the SDI reaches 0.8. Regions such as Tropical Latin America and Western Europe have a higher burden than expected, while regions such as Eastern Sub-Saharan Africa, Andean Latin America, and high-income Asia Pacific have a lower burden than expected. In countries, the burden of migraine in Belgium, Italy, Afghanistan, and Yemen exceeds expectations, while in Peru, Zambia, Singapore, and Ethiopia, the burden is lower than anticipated (Fig. 5 and S17-S21).

## Discussion

In 2015, the United Nations established Sustainable Development Goal 3, which aims to “Ensure healthy lives and promote well-being for all at all ages”, outlining the goal to reduce global population mortality and disease burden by 2030 [24]. Women suffering from migraine, particularly WCBA, are closely linked to this goal, as migraine is the most common and disabling disease in this population, with a higher prevalence of associated complications, leading to a greater overall disease burden [25]. Undoubtedly, a thorough understanding of the prevalence trends of migraines in WCBA is crucial for assessing the potential of achieving the related health goals. However, there is currently a lack of comprehensive literature analyses on the prevalence, incidence, and DALYs related to migraine in this population across different countries and regions globally, with previous studies primarily focusing on specific age groups or countries [16, 26]. Therefore, we believe it is necessary to promptly enhance and update the data on the burden of migraine in the global population of WCBA, enabling policymakers to understand the situation and formulate effective prevention and control strategies. This study is the first comprehensive estimation of the prevalence, incidence, and DALYs of migraine in WCBA over the past 32 years using GBD 2021 data on a global scale.

A significant increase in the cases of prevalent, incident, and DALYs of migraines related to WCBA was reported globally, with a percentage change of 48%, 43%, and 47%, respectively, over the past 32 years. This growth may be associated with the global population increase of 45%. Moreover, the prevalence rates and DALY rates of migraine in WCBA globally increased. Conversely, the incidence rates exhibited a declining trend over time. This result implies that there are more existing patients, and the growth speed of new cases is slowing down, possibly due to the decline in the proportion of WCBA in the global population from 25.57% in 1990 to 24.67% in 2021 [27]. Furthermore, this trend could be attributed to the significant advancements in diagnosis, prevention, and prognosis resulting from the accumulated clinical experience among neurological healthcare professionals over the past 32 years, as well as the development of sophisticated diagnostic and therapeutic technology systems.

In 2021, the highest rates of prevalence, incidence, and DALY were shown in high SDI regions. This is consistent with previous research findings, where countries with higher SDI levels bear a higher burden of migraine disease [28]. The rate trends of prevalence, incidence, and DALYs of migraine among WCBA observed in this study differ from the general assumption regarding disease burden in relation to SDI. In general, higher SDI levels are associated with more robust healthcare systems and higher-quality medical services, resulting in a reduced disease burden. In this study, the highest cases and the most significant growth trends in rates of prevalence, incidence, and DALYs of migraine among WCBA were observed in middle SDI regions. These findings are similar to those studies on the global population of all age groups in the same region, indicating that the burden of migraine among WCBA is highest in this region [26]. The most reasonable hypothesis for this result is that, with the economic development of the middle SDI region, there is an increase in migraine triggers and disease diagnosis reporting among WCBA. However, rapid urbanization and industrialization have accelerated changes in people's lifestyles, including sedentary habits, stress, lack of physical activity, overuse of medications and electronic devices, and deterioration of sleep patterns, exacerbating the incidence of migraine. Furthermore, influenced by the local cultural background and the potential stigmatization of headache disorders, such as migraine among WCBA, indirectly weaken the capacity to control the disease burden through sustained economic growth and social development [29, 30].

Over the past 32 years, the most significant increases in prevalence rates and DALY rates of migraine among WCBA were showed in East Asia and Andean Latin America, which may be attributed to social factors such as industrialization or urbanization, inadequate healthcare services, and poor air quality [31, 32]. The significant variations of the cases of prevalence, incidence, and DALYs of migraine among WCBA across different countries were shown in our study. There is a notable polarization trend, particularly among countries in high SDI regions, with differences reaching up to 16-fold between some nations. Notably, the highest percentage changes in the cases of migraine among WCBA were observed in high-income countries, such as Qatar and the United Arab Emirates consistent with global population trends [33]. The opposite trend observed in a few countries may indicate that healthcare and public health management efforts in this field have achieved positive effects in reducing migraine triggers and improving the disease. Continued monitoring of the trends in these countries may provide insights into global migraine prevention and control strategies. Finally, it is essential to develop targeted prevention and treatment measures based on the individual burden of this disease.

In terms of age patterns, a particularly severe new burden of migraine among WCBA globally was observed in the 15–19 age group in 2021. During this age group, females face challenges such as increased academic pressure and rapid physical and mental development, especially during adolescence and menarche. This highlights the importance of special attention and health management for this age group. A rising trend in the cases of prevalence, incidence, DALYs, and incidence rates of migraine among WCBA over the past 32 years globally with age, with the highest increase observed in the 45–49 age group, particularly in middle SDI regions. Additionally, there is a trend of transition in the proportion of migraine cases among WCBA from younger age groups to older age groups, with the most significant increase observed in the 45–49 age group. This phenomenon may be the result of a combination of biological, genetic, psychological, and social factors. The fluctuation of sex hormones, especially estrogen and progesterone, may play a key role in the onset of migraines in women, with women aged 45–49 experiencing hormonal fluctuations during perimenopause and menopause, which may contribute to the increased frequency of migraine attacks [10, 34]. As WCBA age, they face increasingly complex work and social relationships and may even experience a midlife crisis leading to greater stress. On the other hand, during this time, they must juggle multiple roles, including work, family care, and childcare, necessitating high levels of energy and positive emotions, which may result in fatigue or anxiety, exacerbating the frequency of migraine attacks and complications [35]. Furthermore, women have lower thresholds for stress and pain compared to men, making them more susceptible to migraines. The high prevalence and frequency of migraines in women further perpetuate the ongoing gendered “gendering” of this condition. For example, some pharmaceutical marketing attempts to position migraines as a “women’s disease”, further exacerbating gender bias and disadvantaging female patients seeking help [36]. Furthermore, studies have shown that Headache (specifically migraine) is the main manifestation of the neuro-COVID-19 complex, and COVID-19 seems to enhance existing migraine symptoms and morbidity [37]. Furthermore, the insufficient diagnosis and treatment of migraines globally represent a common issue, leading to an increasing heavy burden of migraines with age. The above-mentioned study emphasizes the need for targeted intervention measures, particularly addressing the most common risk factors among the WCBA population.

Pregnancy is an inevitable concern for WCBA, and the significant burden of severe migraines and associated complications in this population hinders the achievement of the United Nations’ Sustainable Development Goals. Compared to the general obstetric population, pregnant women with migraines are at a higher risk of adverse outcomes for both the mother and the newborn, including GDM, HDP, low birth weight, preterm delivery, and cesarean section, which may be associated with medication use and disease activity [14]. In babies born to women with migraine, the duration of hospitalization was longer, and respiratory problems were more common [38]. Therefore, there is an urgent need for large-scale, evidence-based medicine research on the prevention and treatment of migraine in pregnant women to provide personalized and optimal diagnosis and treatment strategies for clinical physicians. Furthermore, it was striking that almost 20% of women with migraine in the American registry for migraine research database results attested to pregnancy avoidance because of migraine [39]. Therefore, closely monitoring clinical conditions, controlling disease activity, and adjusting medication treatment plans promptly are crucial for women who suffer from migraine before and during pregnancy.

It is worth noting that HC is one of the most commonly used contraceptive methods among WCBA. The use of HC by WCBA can impact the burden of migraine and should be considered in the comprehensive management of migraine in women. However, a survey involving 851 gynaecologists in Germany revealed that they actively consider migraine before and during the prescribing of hormonal contraceptives, and the diagnosis of migraine influences their prescribing behaviour. Although progestogen monotherapy is not associated with additional stroke risk, investigated gynaecologists remained reluctant to prescribe this estrogen-free contraception for migraine with aura. The study also revealed that more than 3/4 of patients with a history of migraine are actively engaged in preventive migraine treatment upon initiating HC, for further migraine treatment the majority of gynecologists would recommend patients refer to a neurologist. This highlights the close overlap between the specialities of neurology and gynecology regarding the patient collective of WCBA suffering from migraine [15]. Another survey involving 115 female healthcare providers revealed that only 6% were aware of migraine treatment guidelines, and only 37% had received headache-specific education, which indicates they appear to have several knowledge gaps in the management of patients with migraine [40]. These may further exacerbate the burden of migraines in WCBA. Therefore, future improving interdisciplinary collaboration between gynaecologists and neurologists may improve migraine treatment for WBCA suffering migraine.

### Limitation

There are several limitations to this study. Firstly, the estimates presented in this paper are not comprehensive as we solely focused on migraines in WCBA as a level four disease rather than level three "headache disorders". Due to lower healthcare standards in some underdeveloped countries, there may be misdiagnosis and underdiagnosis of the condition, leading to an underestimation of the burden. Secondly, the data obtained from the GBD largely relies on modelling data, as GBD collaborators utilize numerous statistical modelling methods, especially in countries where original data is lacking. Thirdly, the burden of migraine is complex to define under one aspect, the disability weights definitions are still very partial as a result, reliable "measurability" of the disability is very difficult. Furthermore, it is important to note the lagged nature of GBD data. Therefore, on the one hand, there is a need for further development of scales and coefficients referring to patients’ disability which might inform future GBD definitions of DW specific to migraine. on the other hand, additional real-world studies are needed to validate the results for a more accurate and comprehensive assessment.

## Conclusions

Overall, the burden of migraine among WCBA has significantly increased globally over the past 32 years. In terms of socioeconomic factors, regardless of the region or specific country, this burden is positively correlated with SDI. The most notable and heaviest disease burden growth was observed in middle SDI regions. This increasing trend may be attributed to unequal opportunities across different regions of the world to access preventive, diagnostic, and treatment resources. In terms of age patterns, the new burden of migraine among WCBA was most severe in the 15–19 age group globally in 2021, while over the past 32 years, the most significant growth in migraine burden occurred in the 45–49 age group. Therefore, urgent interdisciplinary actions involving education, policies, legislation, and medical practices are required to meet Sustainable Development Goal 3 of reducing global population mortality rates and disease burden by 2030. Simultaneously, increasing resource investment in the healthcare of migraine disorders in WCBA, especially during adulthood, is urgently needed to decrease the risk for successively younger birth cohorts and for all age groups over the progressing period.

### Supplementary Information

Supplementary Material 1.

Supplementary Material 2.

## Data Availability

The datasets analysed during the current study are available in the [Global Burden of Disease Study] repository, [https://ghdx.healthdata.org/gbd-2021/sources].

## Abbreviations

WCBA: Women of Childbearing Age
GBD: Global Burden of Disease
HC: Hormonal Contraception
IHME: Institute for Health Metrics and Evaluation
DALYs: Disability-Adjusted Life Years
YLLs: Years of Life Lost
YLDs: Years Lived with Disability
ICHD-3: Classification of Headache Disorders^3rd^ edition
ICD: International Classification of Diseases
UI: Uncertainty Intervals
SDI: Socio-Demographic Index
EAPC: Estimated Annual Percentage Change
CI: Confidence Intervals
GDM: Gestational Diabetes Mellitus
HDP: Hypertensive Disorders of Pregnancy
